# The Effects of Green Intellectual Capital on Green Innovation: A Green Supply Chain Integration Perspective

**DOI:** 10.3389/fpsyg.2022.830716

**Published:** 2022-06-28

**Authors:** Danping Liu, Xiao Yu, Mei Huang, Shaohua Yang, Salmi Mohd Isa, Mao Hu

**Affiliations:** ^1^School of Management, Xihua University, Chengdu, China; ^2^Research Institute of International Economics and Management, Xihua University, Chengdu, China; ^3^Graduate School of Business, Universiti Sains Malaysia, Penang, Malaysia

**Keywords:** green innovation, green intellectual capital, green supply chain integration, relationship learning ability, green absorptive ability

## Abstract

To demonstrate how green innovation (GI) effectively occurs, this study examines the effects of green intellectual capital (GIC) on GI from the perspective of green supply chain integration (GSCI). Based on a natural-resource-based view and knowledge-based view, the authors constructed an intermediary model of GIC-GSCI-GI, and analyzed the effects of green absorptive ability (GAA) and relationship learning ability (RLA) as moderators. An empirical survey of 328 Chinese manufacturing companies was conducted. Our results indicate that three dimensions of GIC positively impact GI. The mediating effects of internal and external GSCI exist in the relationship between GIC and GI. The moderating effects of GAA and RLA in these effects were also verified. Our study provides further empirical evidence for the relationship between GIC and GI, highlights the effects of companies’ internal and external abilities on GI, and suggests new ways and implementation contexts for GI.

## Introduction

With the rapid development of China’s industry, China’s manufacturing share has catapulted to a leading global position. But large-scale industrial operations and massive production expansions have generated increasingly serious ecological and environmental problems which threaten the continued sustainable development of the manufacturing sector (Sun et al., 2020). Hart (1995) constructed a sustainable development model based on natural-resource-based view in which organizations implement proactive environmental strategies to protect the environment and prevent pollution caused by manufacturing activities rather than reactive activities that merely comply with existing environmental regulations (Francesca, 2019; Barrena-Martínez et al., 2020). Researchers suggest that one of the best mechanisms for firms to proactively deal with environmental issues is innovation (Schoonhoven et al., 1990; Tang et al., 2018; Fu et al., 2021). Furthermore, as awareness of environmental degradation grows, consumers are becoming more willing to pay for green products to help protect the planet (Yabar et al., 2013; Barrena-Martínez et al., 2020). According to a 2018 consumer survey in Beijing, Shanghai, Tianjin, Guangzhou, and Harbin by the Social Survey Institute of China, more than 50% of consumers buy green products (Wong et al., 2020). Thus, many organizations have adopted green innovation (GI) strategies to achieve environmental protection and economic growth (Takalo et al., 2021).

Green innovation is defined as the action of integrating green concepts (e.g., environmentally friendly techniques) into business operations (Triguero et al., 2016; Barrena-Martínez et al., 2020). It includes implementing new ideas and/or new methods with respect to products and services, organizational structures, production processes, skills, etcetera, to reduce the negative effects of the production life cycle and to increase economic and environment benefits (Chen, 2011; Arfi et al., 2018; Zhang et al., 2019). Hence, understanding how GI effectively occurs has become critical for many organizations.

Researchers have identified several factors that drive GI, including market demand (Zailani et al., 2015; Kunapatarawong and Martínez-Ros, 2016), the influence of stakeholders (Yabar et al., 2013; Ebrahimi and Mirbargkar, 2017; Borsatto and Amui, 2019; Lee, 2020), skill (Amore and Bennedsen, 2016; Triguero et al., 2016; Albort-Morant et al., 2018a; Kovacova and Lăzăroiu, 2021), collaboration and networking (Cai and Zhou, 2014; Triguero et al., 2016), organizational structures, and social, cultural and ethical factors (Yabar et al., 2013; Chiarvesio et al., 2015; Huang and Li, 2017). Although many studies have examined the antecedents of GI, little attention has been given to green resources and capabilities related to environmental management and the source of GI, green intellectual capital (GIC).

Based on a knowledge-based view, researchers deem that firms should be viewed as knowledge pools, and knowledge needs to be managed for maximum return (Grant, 1997; Abel and Seng, 2020). Chen (2008) argued that factors and capabilities relative to knowledge, intangible assets, and intellectual capital are leading drivers of environmental innovation. Indeed, intellectual capital is the principal basis of business innovation (Chang and Chen, 2012; Yabar et al., 2013; Yusliza et al., 2019). Thus, the process of accumulating GIC is, in fact, a process of business value creation. However, it remains unclear how best to promote the transformation of GIC into GI. Using GIC as an independent variable, prior research has identified the positive effects of GIC on GI (Andersén, 2021). According to Chen (2008), GIC can be categorized as green human capital (GHC), green structural capital (GSC), and green relational capital (GRC), but research about the impacts of these three dimensions on GI and how these impacts work is limited (Yusliza et al., 2019; Wang et al., 2020). Research on the effect of GIC should objectively consider how to activate the three dimensions of GIC flexibly in order to gain competitive advantage, create value, and achieve maximum economic and environmental performance.

In addition, as environmental pollution is often embedded in supply chains, GI requires the involvement of supply chain partners and a great number of resources (Tukamuhabwa, 2011; Wang et al., 2020). Hence, cooperation is essential throughout the supply chain (Valderrama et al., 2020). Furthermore, rapid technological advancements, competitive pressures, and market uncertainties can challenge companies’ GI accomplishments, overwhelm their efforts (Yusliza et al., 2019), and compel them to seek support from both internal and external sources. With involvement throughout supply chain, GI becomes more effective, less risky and less costly as inputs and research expenses are reduced (Afshari et al., 2019; Li et al., 2020c). Green supply chain integration (GSCI) plays an indispensable role in the relationship between GIC and GI, and supply chain integration, including information sharing and collaboration with supply chain members, may help companies to realize GI.

In the context of GSCI, GI performance varies according to a company’s internal and external capabilities, including green absorptive capability (GAC) and relationship learning capability (RLC). Studies have shown that absorptive capability promotes innovation (Soo et al., 2017), performance (Kostopoulos et al., 2011), knowledge transformation within firms (Sözüer and Semerciöz, 2016), and intra-organizational learning activities (Galindo-Martín et al., 2020). In addition, Lee and Humphreys (2007) demonstrated that relationship learning capability has a positive impact on a firm’s knowledge acquisition and promotes new knowledge generation. Such research provides insight into the impact of both internal and external impacts on the relationship between cooperative activities and GI. We believe that the moderating role of these internal and external capabilities on GSCI, and thereby on GI, is important to the extension of GIC — GI advancement.

Although previous studies have identified positive effects of GIC on GI (Andersén, 2021), the three dimensions of GIC have been largely overlooked. By disregarding the relationship between an enterprise and its entire supply chain, including both internal and external capabilities, researchers have failed to generate comprehensive understandings about the effects of GIC on GI. To fill this research gap, we propose an integrated conceptual framework among GIC, GSCI, and GI that incorporates companies’ internal and external capabilities.

The aim of this study is to explore how GIC affects GI from the perspective of GSCI. Firstly, in this research the effect of GIC on GI is explored in three dimensions (GHC, GSC, and GRC) rather than as a single variable as in prior research. Secondly, based on the natural-resource-based view and the knowledge-based view, we developed a conceptual framework by proposing GSCI as a mediator in the causal continuum of GIC to GI. Finally, both internal and external capabilities of companies (namely GAC and RLC) are proposed as moderators to investigate the boundary conditions of the GSCI mechanism in this causal continuum. In most cases, previous research considers one of the two abilities as antecedent variables of GI; however, this study extends the research context by examining the moderating effects of both sources of capability. Thus, the results in this research offer specific operational recommendations for GIC management.

The remainder of this paper is organized as follows. Section “Theoretical framework and hypotheses” reviews the theoretical framework that forms the basis of this empirical analysis. Section “Materials and Methods” describes the study’s research methodology. Section “Results” presents the data analysis results. Section “Discussion” discusses the research findings. Finally, Section “Conclusion” elaborates on the implications of the research findings, limitations, and future research.

## Theoretical Framework and Hypotheses

### Natural-Resource-Based View

The resource-based theory argues that valuable, inimitable firm resources and capabilities contribute to sustainable competitive advantage (Perterlaf, 1993). Extending the resource-based view, Hart (1995) proposed the natural-resource-based view and highlighted the influence of organizational capabilities on an enterprise’s environmental strategy, which in turn provides a competitive advantage for the enterprise.

The literature on natural-resource-based view (NRBV) explores various environmental capabilities such as shared vision (Alt et al., 2015), strategic proactivity (Sharma et al., 2007), innovation (Sharma and Vredenburg, 1998), cross-functional integration, stakeholder engagement, organizational learning and more (Yang et al., 2019). Among these capabilities, innovation can create higher value through increased revenue, greater stakeholder value, improved corporate image, and even improved “green” performance (Baker and Sinkula, 2005), given that “greenness” is a driving force for continuous innovation and value creation (Schiederig et al., 2012). Hence, a natural-resource-based view that considers environmental issues has frequently been used to assess innovative activities and has provided the theoretical foundation for identifying an enterprise’s internal resources that foster environmental performance (i.e., GI, Hart and Dowell, 2010). Furthermore, Seman et al. (2012) argue that GI is the foundation of green supply chain management. When companies are closely integrated together with their suppliers and customers, their GI capabilities are enhanced (Chiou et al., 2011; André and Barbieri, 2012). Thus, GSCI may play an indispensable role in the incentive mechanism of organizational GI.

### Knowledge-Based View

Knowledge-based resources are ways in which firms combine and transform tangible input resources (Galunic and Rodan, 1998). They are vitally important for providing a sustainable competitive advantage (McEvily and Chakravarthy, 2002). Based on the knowledge-based view, Chen (2008) developed the construct of GIC in association with the trends of international environmental regulations and the environmental consciousness of consumers, and defined it as the sum total of all intangible assets, knowledge, capabilities, and relationships in the field of environmental protection at the individual level and the organizational level within a company. It has been demonstrated that GIC has a positive impact on firms’ competitive advantage (Chen, 2008; Bootz et al., 2019).

Effective knowledge creation is now a top priority in supply chains (Wu, 2008). In order to create new knowledge, supply chain partners engage in interlinked processes that enable rich information sharing and build information technology infrastructures that allow them to process the information obtained from partners (Seggie et al., 2006). An organization’s ability to continuously absorb, disseminate, and utilize new knowledge is widely recognized as an important determinant of sustainable competitive advantage (Lane et al., 2006). On the other hand, companies can integrate their supply chain network relationships to generate internal collaboration opportunities (Carter et al., 2007) and to galvanize management improvements. Hence, organizations can use their intellectual capital to integrate their supply chains and carry out innovation activities that enhance sustainable competitive advantage.

### Green Intellectual Capital and Green Innovation

Researchers have begun to pay increased attention to the management of GIC recently (Benevene et al., 2021). Based on the definitions proposed by Chen (2008), López-Gamero et al. (2011), and Chang and Chen (2012), GIC refers to the sum of various intangible assets including knowledge, information, technology, and environmental protection and green management abilities, which create value and provide sustainable competitive advantages for enterprises.

With the advent of the knowledge economy era, intangible assets play an increasingly important role in securing and maintaining competitive edge. According to Chen (2008), GIC can be classified into three categories: GHC, GSC, and GRC. Researchers have begun to study innovation taking these three components of GIC as critical antecedent factors (Li et al., 2020a).

Green human capital refers to the accumulation of general environmental knowledge and ability of employees and their commitments (Chen, 2008). The reserve and development of GHC facilitates the sustainable development of new ideas, products, services and processes which reflect green values (Yusliza et al., 2019). GHC is embedded in a company’s employees and managers but not the framework of the organization, and it can dissipate when employees leave (Kunapatarawong and Martínez-Ros, 2016). The environmental knowledge and abilities of employees and managers play an important role in GI and environmental management in the face of external pressures (Wang et al., 2020). Additionally, employees’ innovative mindsets are critical sources of GI (Yusliza et al., 2019). Hence, in the context of the green economy, human resources with green knowledge, technology, and creativity are fundamental to GI.

Green structural capital refers to reserves of organizational capabilities, organizational commitments, knowledge management systems, reward systems, information technology systems, databases, managerial operating processes, managerial philosophies, organizational culture, etcetera, related to a company’s environmental protection behaviors (Chen, 2008). The core idea of GSC is to reposition and rethink existing organizational structure and business processes congruent with environmentally friendly principles in order to cultivate new organizational structures that align with the dynamics of the environment, thus promoting GI (Jirakraisiri et al., 2018). An enterprise’s cultural atmosphere also has a positive effect on corporate innovative behavior (López-Gamero et al., 2011; Barrena-Martínez et al., 2020). Additionally, GSC, including rationally designed internal organizational structures, sound environment management systems, information technology systems, and the entire operational process, together with rewards systems and a green informant database, all bring incremental value and have positive effects on GI.

Green relational capital refers to interactive relationships between a company and its customers, suppliers, and partners with respect to environmental protection and green management issues (Chen, 2008). Hence, GRC is closely related to a firm’s social relationships. Specifically, it refers to informal personal social relationships built by management and staff. These relationships depend on environmental commitment and cooperation and are not predetermined by the firm (El-Kassar and Singh, 2019). When enduring interactive relationships with customers, suppliers, and partners are established, communication, information sharing and resource exchanges are facilitated, thus bolstering GI awareness and GI efficiency (Wang et al., 2020). Therefore, the enhancement of innovative capabilities and development of green products and technologies are, in large part, reliant on relationships with consumers, suppliers, and even competitors, in that reserves of GRC positively impact GI. Based on the preceding discussion, it is hypothesized that:

H1a: *Green human capital has a positive effect on GI.*

H1b: *Green structural capital has a positive effect on GI.*

H1c: *Green relational capital has a positive effect on GI.*

### The Relationship Between Green Intellectual Capital and Green Supply Chain Integration

Green supply chain integration can be defined as “the collaboration of a firm with its supply chain partners to manage both intra- and inter-organizational environmental practices” (Flynn et al., 2016). Hence, GSCI is responsible for effectively organizing resources derived from both inside and outside a company. Accordingly, GSCI can be divided into internal and external GSCI (Wu, 2013; Lii and Kuo, 2016). Internal GSCI focuses on removing cross-functional barriers, enhancing firms’ environmental initiatives, and motivating employees to learn environmental protection measures (Lo et al., 2018). Wu (2013) extended the concept of GSCI and redefined external GSCI as collaboration activities, including environmental practices focused on energy usage, material consumption, emissions, and waste in connection with in-house processes, with suppliers and customers on environmental practices.

The effectiveness of internal GSCI is improved with high GIC. Firstly, highly qualified GHC provides GSCI performers with more and better environmental knowledge. This is because employee capabilities and commitments to environmental activities enable every phase of the production process to meet environmental standards (Wong et al., 2020). Additionally, mid-to-senior level managers become more supportive of inter-departmental cooperation, eliminating barriers and enhancing knowledge sharing between departments (Albort-Morant et al., 2016).

Secondly, GSC reinforces internal GSCI. This is because, through the enhancement of organizational ability and commitment of environmental management and protection, internal communication is reinforced and intra-departmental cooperation enhanced (Song et al., 2017). Furthermore, the implementation of environmental regulatory regimes, knowledge management systems, databases, compensation systems, and information technology systems serve as platforms for cross-departmental and cross-functional cooperation systems (Flynn et al., 2016).

Thirdly, GRC supports internal GSCI because good relationships between external network members (i.e., supply chain partners, customers, and other strategic partners) fosters a cooperative atmosphere within the company, which leads to improved cross-departmental communication and willingness to accumulate and share information regarding environmental issues, thereby further facilitating the development of seamless environmental management systems throughout the supply chain (Pihlajamaa et al., 2017; Li et al., 2020b). Therefore, the relationships between GIC and internal GSCI are hypothesized as shown below:

*H2a: Green human capital has a positive effect on internal GSCI.*

*H2b: Green structural capital has a positive effect on internal GSCI.*

*H2c: Green relational capital has a positive effect on internal GSCI.*

Meanwhile, high GHC has a positive effect on external GSCI. Customer integration is activated by qualified staff who disseminate environmental knowledge and share techniques, scientific findings and development plans with customers (Zhao et al., 2011). Through their interactions with stakeholders, these individuals also serve as feedback channels, thus facilitating two-way communication and nurturing customer satisfaction with respect to firms’ environmental practices (Lo et al., 2018). The involvement of well-trained employees and managers in purchasing and operational processes reduces production risk by helping suppliers comply with environmental requirements, sharing green information, and discussing green manufacturing technologies (Albort-Morant et al., 2016).

High GSC is also beneficial for external GSCI. Normally, a hybrid structure that includes environmental cooperation and supervision provides auditing of the implementation of environmental management activities by suppliers (Tariq et al., 2017). A company’s green culture and image contributes to environmental thinking, planning, and communication with suppliers and consumers (Song et al., 2017). The properties of green trademarks, patents, and green products tend to attract like-minded suppliers and consumers and help build partnerships in the environmental domain (Mikalef et al., 2019).

Finally, high GRC is also beneficial for external GSCI because relationships of trust among firms, consumers and suppliers make them more amenable to participating actively in environmental problem solving, such as setting environmental goals, discussing green product specifications, researching clean technologies, negotiating environmental strategies about pollution reduction and using green packaging (Lii and Kuo, 2016). Therefore, the relationships between GIC and external GSCI are hypothesized as below:

*H3a: Green human capital has a positive effect on external GSCI.*

*H3b: Green structural capital has a positive effect on external GSCI.*

*H3c: Green relational capital has a positive effect on external GSCI.*

### The Relationship Between Green Supply Chain Integration and Green Innovation

Innovation is motivated by internal integration. Through internal integration, in-house design, manufacturing and distribution departments can cooperate freely in support of product design and production process improvements (Wong et al., 2013; Yusof et al., 2016), and GI becomes embedded in every step of product design and manufacturing (El-Kassar and Singh, 2019). GI depends heavily on the support of mid-to-senior level department managers. As support from mid-to-upper-level managers on environmental issues and sustainable development is essential for goal setting and reward systems, this in turn increases employee motivation to go green. Internal integration also enhances cooperation and alignment on environmental protection practices and environmental knowledge accumulation, as well as environmental management system implementation across departments (Lii and Kuo, 2016). These activities help firms maintain balance between ecological benefits and profitability, in support of GI (Tariq et al., 2017). Therefore, we posit that internal GSCI, which includes managerial support, cooperation among departments and establishment of an environmental management system, promotes GI.

Supply chain integration is an interactive process (Li et al., 2020b). Wong et al. (2020) found that information sharing and collective development of the supply chain base are advantages of external integration. For GSCI, the main features of supplier and consumer integration are green knowledge sharing and active environmental problem solving (Seman et al., 2012; Tariq et al., 2017), including exchanging knowledge about green techniques and green material management. Features specific to supply chain partners include exploring environmental issue-related methods and pursuing implementation of environmentally acceptable ecological designs (Gallardo-Vázquez et al., 2019). By integrating suppliers and consumers, a comprehensive environmental management plan can be implemented, making it more practical for companies to develop and use recycled or less environmentally harmful inputs in production as well as to redesign or adopt manufacturing techniques to reduce waste (Wang et al., 2020). Therefore, companies’ GI abilities are enhanced by close integration with suppliers and consumers, and we hypothesize as below:

H4a: *Internal GSCI has a positive effect on GI.*

H4b: *External GSCI has a positive effect on GI.*

### The Mediating Role of Green Supply Chain Integration

To the authors’ knowledge, there is no research using GSCI mediators to explore the mechanism of GIC on GI. But it is well known that it is difficult to achieve GI based exclusively on the internal resources of single company since environmental degradation is attributable not to any one company but to industry more broadly (Wong et al., 2020). GIC, which is considered one of a company’s most critical resources, is also an important resource in the supply chain (Mikalef et al., 2019). And it is the most direct way to implement supply chain resource integration to convert GIC into GI. Based on prior studies, both internal and external GSCI are integral to firms’ competitive advantage and bring both economic and non-economic benefits. Hence, both internal and external GSCI are helpful for turning GIC into GI.

Internal GSCI refers to the extent of cross departmental cooperation and communication regarding environmental matters within a firm. Close cooperation and seamless communication, including cooperation between product design, purchasing, selling, and marketing departments, as well as activities involving learning systems, are strengthened by staff who possess and accumulate green knowledge (GHC). A green organizational culture and an environmentally friendly organizational structure (GSC) further reinforce internal GSCI (Wong et al., 2020). Finally, there are positive impacts of GRC on internal GSCI. In addition to promoting the effects of internal GSCI on GI, internal GSCI may mediate the impact of GIC on GI.

Suppliers and consumers both rely on supply chains to be profitable, and hence sustainable, and also to generate sustainable development (Yusof et al., 2016). As discussed previously, there are positive impacts from GHC, GSC, and GRC on external GSCI, that, together with effective external GSCI, can earn the satisfaction and trust of suppliers and consumers and also help achieve higher GSCI operational effectiveness, thereby enabling companies to enhance their GI capabilities (Zhao et al., 2011; Wong et al., 2020). Thus, we posit that external GSCI mediates the impact of GIC on GI. The relative hypotheses proposed are below:

*H5a: Internal GSCI positively mediates the relationship between GHC and GI.*

*H5b: Internal GSCI positively mediates the relationship between GSC and GI.*

*H5c: Internal GSCI positively mediates the relationship between GRC and GI.*

*H6a: External GSCI positively mediates the relationship between GHC and GI.*

*H6b: External GSCI positively mediates the relationship between GSC and GI.*

*H6c. External GSCI positively mediates the relationship between GRC and GI.*

### The Moderating Role of Relationship Learning Ability and Green Absorptive Ability

Relationship learning emphasizes the ability to learn external behaviors through communication, adjustment and cooperation between upstream and downstream enterprises, so as to promote joint actions among alliance partners, suppliers, and channels (Capello and Faggian, 2005). Through relational learning, enterprises can enhance information exchange between their upstream and downstream enterprises, refresh their R&D capabilities, and create greater value than as individuals (Albort-Morant et al., 2018b). Based on research by Selnes and Sallis (2003), we define RLA as the effectiveness of a company’s learning activity improvement through information exchange, communication, common learning field development, and frequent interactions with suppliers, customers, cooperation partners, and other stakeholders. In these ways, a company learns from environmental information provided by suppliers and consumers how to better implement their environmental management plans, utilizing methods such as developing recyclable materials and redesigning wasteful production processes (Jean et al., 2010; Kohtamäki and Partanen, 2016; Albort-Morant et al., 2018b).

Companies are motivated to launch learning activities to enhance performance and boost competitiveness. By establishing learning relationships, companies can acquire environmental information from suppliers and customers to leverage the effects of external cooperation and communication activities on innovative performance (Sözüer and Semerciöz, 2016). Although some previous research has investigated the positive impacts of learning ability on innovation, we specifically argue that a high (vs. low) RLA causes GSCI to have to a stronger (vs. weaker) impact on GI (Slåtten et al., 2017). The moderating role of RLA is hypothesized below:

H7: *Relationship learning ability positively moderates the effect of external GSCI on GI. Companies with higher (vs. lower) RLA show better (vs. worse) GI performance through external GSCI.*

When it comes to a firm’s internal aspects, effective internal management is needed to motivate employees and boost innovation. Adopting the definition of Chen et al. (2015), GAA is the ability to understand, communicate, combine, identify and commercialize environmental knowledge. A company’s absorptive ability is connected to its R&D resources, communication mechanisms and administrative processes (Albort-Morant et al., 2018b). Companies with high (vs. low) GAA can take more (vs. less) advantage of recognition and accessibility of critical external green knowledge and information, enhance evaluation and predictive abilities based on this information, activate green knowledge implementation techniques and close cooperation across functional departments, and eventually improve (vs. weaken) GI. Therefore, we propose the following hypothesis:

H8: *Green absorptive ability positively moderates the effect of internal GSCI on GI. Companies with higher (vs. lower) GAA gain better (vs. worse) GI performance by internal GSCI.*

**FIGURE 1:**
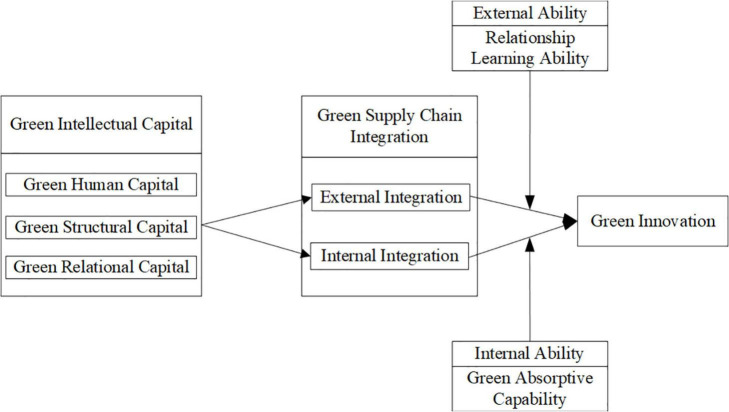
Framework of the research.

Figure 1 displays the research framework.

## Materials and Methods

### Participants and Procedures

This study focused on companies that have a significant impact on the natural environment and have implemented environment-related actions over the past two years. According to the 2010 “Guide for Environmental Information Disclosure of Listed Companies” produced by the Ministry of Environmental Protection, PRC, heavy polluting industries are: mining, food, beverages, textiles, clothing, fur, paper-making, printing, petroleum, chemicals, rubber, plastics, electronics, metals, non-metals, pharmaceuticals, biological products, fermentation, steel, electricity, coal, metallurgy and water (He et al., 2016). To gain adequate insight into the marketing, operations and environmental management issues of enterprises in these industries and to ensure that respondents could fully understand and answer the questions posed, respondents in this study were mid- and upper-level managers who had influence on the enterprise, including CEOs, board chairs, and heads of R&D, marketing, production and/or environmental departments.

Between September and October 2017, we distributed 3,587 copies of our questionnaire on a Chinese online platform, wenjuanxing^1^, and 402 were returned. Excluding 74 unqualified responses, 328 valid responses were considered in our study, for an efficiency rate was 81.6%. Table 1 shows basic information about the sample.

**TABLE 1:** Results of descriptive analysis of samples.

Variable	Category	Frequency	Percentage
Manager position	CEO/general manager	15	4.6
	Corporate legal person	29	8.8
	Chairman	26	7.9
	R&D manager	22	6.7
	Marketing manager	52	15.9
	Producing manager	55	16.8
	Environment, health and safety leader	28	8.5
	Others	101	30.8
Enterprise establishment years	<3 years	7	2.1
	4–8 years	59	18
	9–13 years	133	40.5
	>13 years	129	39.4
Staff size	<100	25	7.6
	101–300	121	36.9
	301–500	58	17.7
	501–1000	48	14.6
	1001–3000	44	13.4
	>3000	32	9.8
Nature of property right	State owned and state holding enterprise	58	17.7
	Private enterprise	221	67.4
	Sino-foreign joint venture	33	10.1
	foreign-owned enterprises	16	4.8
Industry	Mining	7	2.1
	Food, Beverage	27	8.2
	Textile, clothing, fur	24	7.3
	Paper-making, printing	11	3.4
	Petroleum, chemical, rubber, plastics,	40	12.2
	Electronics	46	14
	Metals, no-metals	20	6.1
	Machinery, equipment and instrument	95	29
	Pharmaceutical, biological products	7	2.1
	Production and supply of electricity, gas and water	14	4.3
	Others	37	11.3
Be listed or not	Yes	70	21.3
	No	258	78.7

### Measures

In order to ensure the validity and reliability of our measurement tools, we selected scales that had been validated in previous research and adopted the “reverse translation method” for translation based on the actual situation of Chinese companies (McGorry and Hsiang, 2000). Appropriate modifications were made according to the purpose of this study to form the initial scale. The mid- and upper-level managers of five companies in five different industries (cosmetics, mechanical equipment, clothing, construction, and electrical power) in Sichuan were selected for in-depth interviews to ensure that the expression of the questionnaire accorded with the situation of these Chinese companies (He et al., 2016). Relevant academics and doctoral students were invited to read the questionnaire items and give feedback to help improve these items. Prior to our formal investigation, a small-scale pre-test was carried out. From the results of this, each measurement item was evaluated and modified again, and eventually a final questionnaire was formed.

First, the measures of GIC further include two level indicators. GHC, GSC, and GRC are the second-level indicators adopted from Chen (2008), Chang and Chen (2012), and Yusliza et al. (2019). Specifically, the third-level indicators included five items for GHC, nine items for GSC, and five items for GRC. Second, we adapted an eight-item scale from Chen (2008), Albort-Morant et al. (2016), and El-Kassar and Singh (2019) to measure GI, four items for green product innovation and four items for green process innovation, respectively. Third, internal GSCI was measured with six items, following previous studies (Wu, 2013; Abdullah et al., 2017), while external GSCI was measured with eight items, again following previous studies (Wong et al., 2013, 2020). Finally, RLA was measured with five items (Selnes and Sallis, 2003; Albort-Morant et al., 2016), and GAA was measured with another five items, again following previous studies (Chen et al., 2015; Soo et al., 2017). Details of the questionnaire can be seen in Table 1. We also included four firm characteristics as control variables: scale, nature, age and state of being, as listed in Table 2.

**TABLE 2:** Descriptive statistics and correlation matrix.

Variables	1	2	3	4	5	6	7	8	9	10	11	12
(1) GI	**0.727**											
(2) GHC	0.430**	**0.792**										
(3) GSC	0.515**	0.573**	**0.709**									
(4) GRC	0.465**	0.394**	0.491**	**0.791**								
(5) Internal GSCI	0.444 **	0.351**	0.400**	0.533**	**0.748**							
(6) External GSCI	0.548 **	0.378**	0.464**	0.528**	0.522**	**0.709**						
(7) GAA	0.531 **	0.455**	0.428**	0.517**	0.533**	0.496**	**0.798**					
(8) RLA	0.414 **	0.405**	0.467**	0.387**	0.269**	0.398**	0.372**	**0.791**				
(9) Age	–	0.173**	0.110*	0.072	0.008	0.094	0.081	0.071	–			
(10) Staff Size		0.050	0.077	0.017	0.076	0.039	0.014	0.014	0.439**	–		
(11) Nature		0.105	0.140*	0.035	0.027	0.018	0.013	0.074	0.253**	0.0297**	–	
(12) Be listed or not		0.008	0.021	0.013	0.040	0.101	0.041	0.070	0.104	0.371**	0.129*	–
Mean	3.948	3.921	3.755	3.852	3.688	3.739	2.205	2.149	3.170	3.190	0.180	1.790
SD	0.605	0.625	0.615	0.637	0.585	0.657	0.662	0.717	0.795	1.486	0.382	0.410

*Bold figures on the diagonal are the square root of the AVE for the constructs.*

*p < 0.05; **p < 0.01.

We conducted confirmatory factor analyses (CFA) to test the reliability and validity of our scales. The results indicated a good fit. χ2/df = 2.16; RMSEA = 0.060, CFI = 0.938, NFI = 0.934. In addition, we calculated Cronbach’s alpha and composite reliability for all constructs. The Cronbach’s alpha ranges from 0.834 to 0.855, while composite reliability ranges from 0.884 to 0.901, indicating satisfactory reliability.

The factor loading of all the construct items ranged from 0.662 to 0.844, and the average variance extracted (AVE) of all constructs ranged from 0.503 to 0.637, as listed in Table 3. In addition, the square root of each construct’s AVE exceeded the correlations between the construct and other constructs, indicating satisfactory validity.

**TABLE 3:** Reliability, internal consistency and convergent validity.

Variables	Cronbach’s α	CR	AVE
(1) GI	0.866	0.887	0.529
(2) GHC	0.849	0.894	0.628
(3) GSC	0.842	0.901	0.503
(4) GRC	0.851	0.893	0.626
(5) Internal GSCI	0.834	0.884	0.560
(6) External GSCI	0.843	0.890	0.503
(7) GAA	0.855	0.898	0.637
(8) RLA	0.849	0.893	0.625

*CR means composite reliability and AVE means average variance extracted.*

To examine common method bias (CMB), Harman’s single factor testing was conducted (Podsakoff and Organ, 1986). The results showed that the total variance explained by five factors was 65.656%. The first factor accounted for 28.64% of the variance, which was less than 50%, suggesting that common method bias is minimal. Moreover, the highest correlation coefficient was 0.573, indicating a comparatively low collinearity.

## Results

### Hypothesis Testing

The principal effects of GHC, GSC, and GRC on the GI of enterprises were verified by multiple regression. Table 4 shows significant effects of GHC, GSC, and GRC on GI (*b*_GHC_ = 0.412, *p* < 0.01; *b*_GSC_ = 0.523, *p* < 0.01; *b*_GRC_ = 0.421, *p* < 0.01), indicating that GHC, GSC and GRC promote GI; thus H1-a, H1-b, H1-c are supported.

**TABLE 4:** Results of the regression analysis (direct effects).

Variables	Green innovation							Internal integration				External integration			
	M1-1	M1-2	M1-3	M1-4	M1-5	M1-6	M1-7	M2-1	M2-2	M2-3	M2-4	M3-1	M3-2	M3-3	M3-4
Constants	1.956***	1.816***	1.628***	0.892***	1.852***	1.966***	1.229***	1.716***	1.421***	1.057***	0.468	2.541***	1.877***	2.402***	1.450***
Enterprise establishment years	0.064	0.094**	0.092**	0.093**	0.113**	0.088**	0.113***	0.021	0.053	0.046	0.020	–0.029	–0.013	–0.004	–0.029
Staff size	0.012	0.010	–0.002	0.005	0.020	–0.006	0.004	0.065	0.036	0.065	0.026	–0.032	–0.032	−0.043*	–0.033
Nature of property right	–0.128	–0.092	−0.178**	−0.176**	–0.109	–0.075	–0.109	–0.031	–0.058	–0.014	–0.076	0.037	0.061	0.004	0.027
Be listed or not	0.056	0.077	0.026	0.095	0.061	–0.026	0.038	0.121**	0.103*	0.135*	0.191**	0.009	0.029	–0.013	0.013
GHC	0.412***			0.137***				0.374***			0.106	0.336***			0.109**
GSC		0.523***		0.304***					0.526***		0.230***		0.389***		0.125**
GRC			0.421***	0.240***						0.462***	0.395***			0.491***	0.392***
Internal GSCI					0.413***		0.241***								
External GSCI						0.461***	0.378***								
*R* ^2^	0.188	0.207	0.281	0.365	0.172	0.253	0.357	0.158	0.230	0.298	0.356	0.134	0.293	0.171	0.329
ADJ-*R*^2^	0.175	0.195	0.270	0.352	0.159	0.242	0.345	0.144	0.218	0.287	0.342	0.120	0.283	0.159	0.314
*F*	14.880	16.790	25.190	26.321	13.390	21.840	29.657	12.040	19.239	27.285	25.232	9.948	26.750	13.330	22.364

*p < 0.1; **p < 0.05; ***p < 0.01; N = 328.

As shown in Table 4, GHC, GSC, and GRC positively affected internal GSCI (*b*_GHC_ = 0.374, *p* < 0.01; *b*_GSC_ = 0.526, *p* < 0.01; *b*_GRC_ = 0.462, *p* < 0.01), supporting H2-a, H2-b, H2-c. It can also be concluded from these results that all three GICs were positively related to external GSCI (*b*_GHC_ = 0.336, *p* < 0.01; *b*_GSC_ = 0.389, *p* < 0.01; *b*_GRC_ = 0.491, *p* < 0.01); therefore H3-a, H3-b, H3-c are supported. In line with H4, internal GSCI and external GSCI had a positive relationship with GI (*b*_internal GSCI_ = 0.413, *p* < 0.01; *b*_external GSCI_ = 0.461, *p* < 0.01).

We used SPSS PROCESS to test the mediation effects, following the mediation analysis model proposed by Preacher et al. (2007) and Hayes and Preacher (2014).

Results suggested that the external GSCI does mediate the effect of GHC, GSC, and GRC on GI. All intervals do not include 0, and the mediation effect sizes are 0.067, 0.068, and 0.089, respectively. Therefore, H5-a, H5-b, H5-c are supported.

It also seen in Table 5 that there are significant mediation effects between GHC, GSC, and GRC on GI through internal GSCI. All intervals do not include 0, and the mediation effect sizes are 0.133, 0.141, and 0.182, respectively, thus supporting H6-a, H6-b, H6-c.

**TABLE 5:** Results of the analysis (mediation effects).

Independent variables		Dependent variables		Mediator variables	Standardized coefficients	LLCI	ULCI
GHC	→	External GSCI	→	GI	0.067	0.021	0.121
GHC	→	Internal GSCI	→	GI	0.133	0.073	0.200
GSC	→	External GSCI	→	GI	0.068	0.022	0.126
GSC	→	Internal GSCI	→	GI	0.141	0.084	0.202
GRC	→	External GSCI	→	GI	0.089	0.027	0.155
GRC	→	Internal GSCI	→	GI	0.182	0.115	0.250

**FIGURE 2:**
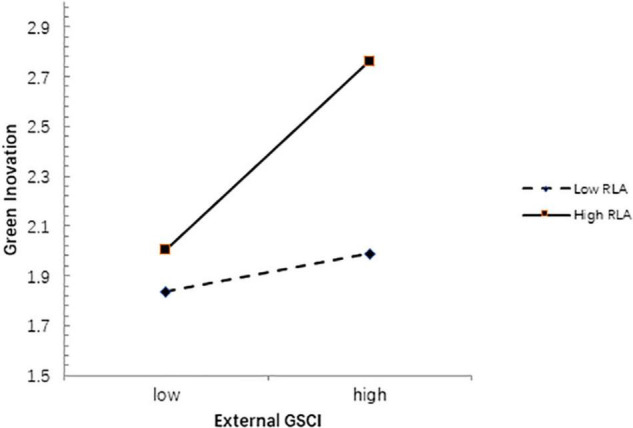
Moderating effect of RLA.

We ran moderated regression models to test the moderating effects. According to the results in Table 6, the interaction between external GSCI and RLA (*b* = 0.211, *p* < 0.01) is significant, indicating that RLA positively moderates the relationship between external GSCI and GI and supporting H7. In addition, we can see that RLA strengthens the positive relationship between the external GSCI and GI, as shown in Figure 2.

**TABLE 6:** Results of the regression analysis (moderation effects).

Variables	Model1	Model2	Model3	Model4	Model5	Model6
Constants	1.808***	1.070***	–1.210**	1.661***	1.130***	–1.132***
Enterprise establishment years	0.181**	0.159**	0.148**	0.091**	0.084*	0.093*
Staff size	0.007	0.026	0.026	–0.023	–0.015	–0.003
Nature of property right	0.009	–0.030	–0.025	–0.061	–0.045	–0.114
Be listed or not	0.070	0.059	0.059	0.052	0.060	0.054
External GSCI	0.370***	0.316***	0.170			
RLA		0.329***	0.436***			
External GSCI * RLA			0.211***			
Internal GSCI				0.521***	0.406***	0.088
GAA					0.251***	0.517***
Internal GSCI * GAA						0.191***
Sample size	328	328	328	328	328	328
*R* ^2^	0.179	0.326	0.359	0.342	0.416	0.475
ADJ-*R*^2^	0.157	0.313	0.345	0.325	0.398	0.455
*F*	20.150 ***	25.834 ***	25.618 ***	19.480 ***	20.070 ***	23.870 ***

*p < 0.1; **p < 0.05; ***p < 0.01. N = 328.

**FIGURE 3:**
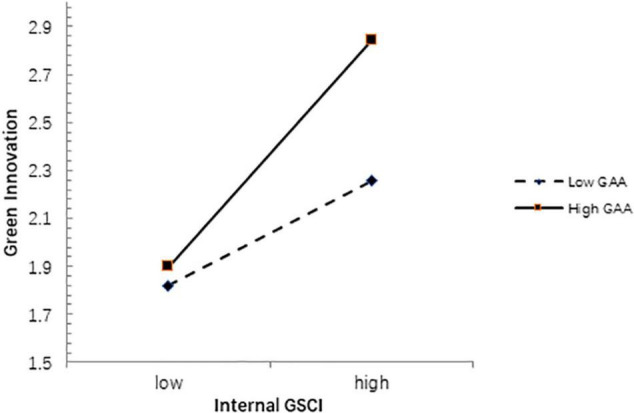
Moderating effect of GAA.

As shown in Table 6, the interaction between internal GSCI and GAA (*b* = 0.191, *p* < 0.01) is significant, indicating that GAA positively moderates the relationship between internal GSCI and GI, so H8 is supported. In addition, we can see that GAA strengthens the positive relationship between internal GSCI and GI, as shown in Figure 3.

## Discussion

With regard to the effects of GIC on GI, our research revealed that all three dimensions of GIC (GHC, GSC, and GRC) had significant positive relationships with GI (H1). Specifically, GHC had a significant positive impact on GI, which is contrary to the finding of Subramaniam and Youndt (2005) but consistent with the research of Andersén (2021). The discrepancy with Subramaniam and Youndt (2005) findings may be attributable to the measure of GI used in this study, which involved the scope of green product and process innovation but not the strength of the innovation (i.e., incremental vs. radical innovation, Subramaniam and Youndt, 2005). Our conclusions about the positive impacts of GSC and GRC on GI are consistent with the results of Subramaniam and Youndt (2005) and Jirakraisiri et al. (2018). Thus, our findings reinforce the idea that GI relies on intangible elements such as employee skills and capabilities, process routines, and networking relations between the firm and its business partners related to environmental issues (Soo et al., 2017; Albort-Morant et al., 2018a).

With respect to the relationship between three dimensions of GIC, both internal and external GSCI and GI, the findings provide empirical evidence that both internal and external GSCI mediated the impacts of GHC, GSC and GRC on GI (H5). Specifically, GHC, GSC, GRC impacted positively on both internal and external GSCI (H2, H3), which impacted positively on GI (H4). And both internal and external GSCI mediated the relationship between the three dimensions of GIC and GI (H5, H6). To our knowledge, this is the first study using GSCI to explain the mechanism of GIC to GI, echoing the research requisite raised by Song et al. (2017). GSCI plays an indispensable intermediary role in the relationship between GIC and GI. Hence, we provide evidence for the bridging effects of internal and external GSCI on the relationship between GIC with GI. Furthermore, RLA and GAA are introduced as moderating variables which influence the relationship between the GSCI and GI (H7, H8). Our results show that an increase in RLA can positively moderate the correlation between external GSCI and GI. These results indicate that high RLA enhances the positive impacts of external GSCI on GI (Sözüer and Semerciöz, 2016) and also that GAA influences the relationship between the internal GSCI and GI. Hence, the premise that high absorptive capability improves the effects of internal GSCI on GI is supported. Taken together, these results further enrich the body of knowledge about the implementation of GSCI for GI.

## Conclusion

### Theoretical Implications

Firstly, our study contributes to the GIC literature by providing further empirical evidence for the effects of GIC on GI. There have been many studies on GI, but specific research on the effects of environmental management-related green resources and abilities, especially concerning the important source of GI: GIC, is limited. Our study divided GIC into three dimensions rather than measuring it as one variable, thus providing more detailed implications for companies seeking to improve GI.

Secondly, our evidence about the mediating effects of internal and external GSCI on the relationship between GIC and GI provides new options for implementing GI. This is one of the few empirical efforts to systematically examine the black box effects of GIC on GI. Prior research has suggested improving GI by GSCI (Song et al., 2017; Li et al., 2020b), but there was little evidence previously to suggest that GSCI could promote GIC boosting GI. Our empirical settings isolated GSCI into internal and external patterns, and thus offered a comparable perspective on assessment of different routes for improving GI performance. Hence, enterprises can choose an optimal way to reap the benefits of GIC on GI performance by GSCI.

Finally, the boundary conditions of the impact of GSCI on GI were further explored in our study, highlighting further the role of companies’ internal and external GI capabilities. As antecedent variables of GI and organizational performance, GAA and RLA have been studied previously (Chen et al., 2009) but with limited focus on possible interaction effects with GSCI on GI. Our evidence sheds light on the varied effects of GSCI on GI performance in the context of firms’ differing GAA and RLA, enriching understanding of GI implementation.

### Managerial Implications

Green development has become an issue of global concern. Faced with increasingly critical environmental issues, GI has become necessary and inevitable. The conclusions of this paper provide key insights for managers seeking to improve GI.

First, corporate leaders are urged to reflect on their companies’ overall relationship with the environment. The clear direct impacts of the three dimensions of GIC on GI suggest that managers should focus on the accumulation of GIC and effectively manage GHC, GSC and GRC to improve GI performance.

Second, we have concluded that both internal and external GSCI significantly mediate the effects of GIC on GI. This finding suggests that when seeking GI improvement through both internal and external resources, it is important to pay attention to integration effectiveness. In other words, both the efficiency of cross departmental and functional cooperation and communication within the company as well as relationships with supply chain partners and consumers are important.

Third, the significant moderating effects of RLA and GAA suggest that it is important for companies to take both capabilities, RLA in the external and GAA in the internal, into consideration. Specifically, companies should construct communication networks, build trust relationships, and foster cooperative atmospheres with suppliers and consumers to increase the effectiveness of external resource utilization. Importantly, companies should also emphasize the speed and capacity to recognize, acquire, evaluate and implement green knowledge and information to enhance the transformation of internal resources to GI.

Finally, governments can construct facilities in industrial areas to reduce waste generated by enterprises. At the same time, governments can also organize conferences or workshops devoted to environmental issues and develop policy to promote GI. Governments can also encourage enterprises to strengthen cooperation with supply chain partners, make more effective use of green resources, and help develop green industries.

### Research Limitations and Future Research

Despite its theoretical contributions and managerial implications, several limitations of this research need to be considered. First, this study derived results using static cross-sectional data, so its reliability may be affected. Because social, economic and corporate development are all fluid, and the relationship between GIC, GSCI, and GI is a dynamic process, it will be fruitful for future research to examine how GIC affects GI over time. This research suggests that future research should use longitudinal or micropanel data to focus on the entire dynamic process.

Second, although this research offers meaningful findings about the relationship between GSCI and GI in China, it may be limited to the institutional and business culture context of China. Future research should examine cross-cultural differences in the relationship between GSCI and GI and include national-level factors such as economic development, social norms, and business cultures in an integrated analysis.

Finally, this study focuses on examining the antecedents of GI. Further research can explore the influencing mechanisms of GI. For example, how would GI affect company culture, leadership, and organizational learning processes at both the individual and team levels? Further exploration and examination of GI influencing factors will help advance the study of the GI mechanism.

## Data Availability Statement

The original contributions presented in this study are included in the article/Supplementary Material, further inquiries can be directed to the corresponding author/s.

## Ethics Statement

Ethical review and approval was not required for the study on human participants in accordance with the local legislation and institutional requirements. The patients/participants provided their written informed consent to participate in this study.

## Author Contributions

DL reviewed the literature, proposed the research model, and designed the study. XY conducted the literature search, proceeded with the data extraction process, and involved in the development of the manuscript. MeH conducted the statistical analysis and revised the manuscript critically for important content. SY revised the whole manuscript according to the comments of the reviewer and rechecked the relevant data of the manuscript. SMI put forward many constructive suggestions on promoting the revision of the manuscript and supervised the entire writing process of the manuscript. MaH participated in the writing of the first manuscript. All authors approved the final manuscript to be published.

## Conflict of Interest

The authors declare that the research was conducted in the absence of any commercial or financial relationships that could be construed as a potential conflict of interest.

## Publisher’s Note

All claims expressed in this article are solely those of the authors and do not necessarily represent those of their affiliated organizations, or those of the publisher, the editors and the reviewers. Any product that may be evaluated in this article, or claim that may be made by its manufacturer, is not guaranteed or endorsed by the publisher.

## Footnotes

1
www.wjx.cn
