# Supplementary material for: The Effects of Green Intellectual Capital on Green Innovation: A Green Supply Chain Integration Perspective
Source: Front Psychol. 2022 Jun 28;13:830716. doi: 10.3389/fpsyg.2022.830716 (PMC9275431; doi:10.3389/fpsyg.2022.830716)
Supplement: Supplementary file 1 [file Data_Sheet_1.docx]

**Appendix. Measurement items and validity assessment**

All items are on a seven-point scale (1 = “strongly disagree,” and 5 = “strongly agree”).

Green Human Capital: To the extents of (CR=0.894, AVE=0.628, Cronbach’s α =0.849)

1. Staffs are superior on environmental productiveness and contribution to main rivals. (0.844)

2. Staffs are superior on environment protection abilities to main rivals. (0.779)

3. Staffs are superior on supply of green production and quality service to main rivals. (0.814)

4. Teamwork of environment protection is superior to main rivals. (0.802)

5. Managers could provide fully support for pursuing environment protection goals. (0.716)

Green Structural Capital: To the extents of (CR=0.901, AVE=0.503, Cronbach’s α =0.842)

1. Environmental management system is superior to main rivals. (0.781)

2. Benefits received from environment protection are superior to main rivals. (0.722)

3. The rate of environment protection R&D inputs on sales revenue is superior to main rivals. (0.700)

4. The capability of green R&D is superior to main rivals. (0.674)

5. The investments on environment protection equipment are superior to main rivals. (0.662)

6. The rate of staffs’ quantity relative to environment protection on total staff is superior to main rivals. (0.683)

7. The whole environment protection operation process is smooth. (0.721)

8. The innovations on environment protection are more than main rivals. (0.717)

9. The environmental management knowledge system conduces to accumulation and sharing of environmental management knowledge. (0.714)

Green Relational Capital: To the extents of the firm is (CR=0.893, AVE=0.626, Cronbach’s α =0.851)

1. Designing products and services according to consumers’ environment protection expectation. (0.766)

2. Satisfaction of consumers on firm’s environment protection behavior is superior to main rivals. (0.799)

3. Having good and solid cooperative relationships with upstream suppliers on environment protection. (0.823)

4. Having good and solid cooperative relationships with downstream suppliers on environment protection. (0.769)

5. Having good and solid cooperative relationships with strategic partners on environment protection. (0.799)

External GSCI: To the extents of the firm is (CR=0.890, AVE=0.503, Cronbach’s α =0.843)

1. Setting environmental goals with suppliers. (0.697)

2. Environmental auditing for suppliers internal management. (0.681)

3. Providing requisition and standards relative to suppliers’ environmental design and clean producing technology. (0.725)

4. Requesting suppliers to implement environmental management or to obtain the third-party certification on environmental management system. (0.689)

5. Selecting suppliers based on environmental standards. (0.689)

6. Co-planning for environmental goals with customers. (0.765)

7. Cooperating with customers to reduce the influence of product on environment. (0.718)

8. Cooperating with customers on clean production, green package, and other environmental protection activities. (0.705)

Internal GSCI: To the extents of (CR=0.884, AVE=0.560, Cronbach’s α =0.834)

1. Middle-senior managers devote to green supply chain management. (0.765)

2. Cross-functional department cooperation is conducted for environmental improvement. (0.720)

3. Cross-departmental communication on environment issues is well conducted within the firm. (0.731)

4. Implementing and auditing projects complying with environmental standards. (0.743)

5. Accumulation and sharing environment protection knowledge between departments. (0.750)

6. Operating an environmental management system. (0.780)

Green Innovation: To the extents of the firm is (CR=0.887, AVE=0.529, Cronbach’s α =0.866)

1. Selection of less / no pollution materials on the phase of product R&D. (0.796)

2. Selection of less energy / resource consumption materials on the phase of product R&D. (0.723)

3. Using the least materials on product R&D. (0.684)

4. Careful consideration on issues of whether products ingredients are recyclable and biodegradable on the phase of product R&D. (0.718)

5. Effectively reducing emission of toxic substances and waste during production process. (0.697)

6. Conduction of recycling, reuse and reproduce of raw material and other waste substances and gas during production process. (0.704)

7. Using clean energies and effectively reducing consumption of resource like water, electric power, coal, and oil during production process. (0.721)

8. Reducing use of raw material as production technology improvement during production process. (0.730)

Relational Learning Capacity: To the extents of the firm is (CR=0.893, AVE=0.625, Cronbach’s α =0.849)

1. Exchanging information related to market structure changes, such as M&A, with cooperation partners. (0.775)

2. Exchanging information related to changes of production technology with cooperation partners. (0.789)

3. Frequent discussion on important strategic issues with cooperation partners. (0.808)

4. Frequently adjusting consensus on business related technology development with cooperation partners. (0.781)

5. Renewing personal social relation networks by frequent communication with cooperation partners. (0.799)

Green Absorptive Capacity: To the extents of the firm is (CR=0.898, AVE=0.637, Cronbach’s α =0.855)

1. Capable on free communication of green knowledge between departments. (0.8290

2. Capable on identification, acquisition, and valuing external green knowledge which has critical impact on firm’s operation. (0.793)

3. Capable on implementing green knowledge effectively on perusing commercial goals. (0.774)

4. Capable on improving analyzing, understanding, and predicting information based on external green knowledge, with the help of rational organization structure. (0.763)

5. Capable on integration of existing green knowledge with newly obtained green knowledge. (0.829)
